# Identification of Endothelial Progenitor Cells in the Corpus Cavernosum in Rats

**DOI:** 10.1155/2014/910564

**Published:** 2014-10-21

**Authors:** Jun Sik Lee, In Sang Hwang, Hyun-Suk Lee, Mi Eun Kim, Young-Woo Seo, Kwangsung Park

**Affiliations:** ^1^Department of Biology, BK21-Plus Research Team for Bioactive Control Technology, College of Natural Sciences, Chosun University, Gwangju 501-759, Republic of Korea; ^2^Department of Urology and BK21 PLUS Center for Creative Biomedical Scientists, Chonnam National University Medical School, Sexual Medicine Research Center, Chonnam National University, 8 Hak-Dong, Dong-Gu, Gwangju 501-757, Republic of Korea; ^3^Korea Basic Science Institute, Gwangju Center, Gwangju 500-757, Republic of Korea

## Abstract

The vascular wall resident progenitor cells seem to serve as a local reservoir of cells for vascular repair. It was hypothesized that the corpus cavernosum may contain vascular wall endothelial progenitor cells (EPCs). In this study, we investigated the identification and localization of EPCs in the corpus cavernosum in a rat model. Adult male Sprague-Dawley rats were used to isolate EPCs from corpora cavernosum. To verify the existence and localization of EPCs, EPC-specific markers (CD34, Flk-1, and VE-cadherin) were evaluated by flow cytometric analysis and confocal microscopy. The EPC markers were mainly expressed in the cavernosal sinusoidal endothelial space. EPC-marker-positive cells made up about 3.31% of the corpus cavernosum of normal rat by FACS analysis. As shown by confocal microscopy, CD34^+^/Flk-1^+^ and CD34^+^/VE-cadherin^+^ positive cells existed in the corpus cavernosum. Our findings imply that regulation of corpus cavernosal EPCs may be a new therapeutic strategy in the treatment of erectile dysfunction.

## 1. Introduction

Penile erection is moderated by vascular, neural, and hormonal factors as well as by cavernosal structural integrity. The cavernosal endothelium and smooth muscle cell compartments have crucial roles in vascular hemodynamics during penile erection. The endothelial monolayer plays a key role in erection physiology; therefore, any cellular or molecular impairment of endothelial cells results in erectile dysfunction [1]. Recent studies have shown that the incidence and development of erectile dysfunction is closely related to the dysfunction of endothelial cells [2].

Endothelial progenitor cells (EPCs) play a major role in new vessel formation. EPCs are a population of rare cells that circulate in the blood with the ability to differentiate into endothelial cells and make up the lining of blood vessels [3]. Furthermore, EPCs can proliferate and are involved in vasculogenesis. Several reports have shown that the number of circulating EPCs is decreased in patients with erectile dysfunction [4, 5]. Recently, vascular wall resident progenitor cells were introduced as a source for postnatal vasculogenesis [6]. These vascular wall resident progenitor cells seem to serve as a local reservoir of cells for vasculogenesis. It has also been reported that osteogenic progenitor cells in human tunica albuginea may originate from stem cells [7]. Furthermore, Traish et al. hypothesized that androgen deprivation promotes the differentiation of progenitor stromal cells into an adipocyte lineage [8].

It was hypothesized that the corpus cavernosum contains vascular wall EPCs. Therefore, we aimed to identify and localize EPCs in the corpus cavernosum in a male rat model.

## 2. Materials and Methods

### 2.1. Animals

Adult male Sprague-Dawley rats (12 weeks old, *n* = 20) were used in the current study. Penile tissue was harvested for biochemical analyses as described below. This study was approved by the Ethics Committee of the Chonnam National University Medical School.

### 2.2. Immunohistochemical Staining

With the rats under ketamine anesthesia, the corpus cavernosum was perfused with cold saline via the abdominal aorta. The penis was then rapidly removed and the corpus cavernosum was carefully dissected away from the urethra and surrounding connective tissue under optical magnification. A midportion of the corpus cavernosum was harvested for immunohistochemical assessment. The cavernous tissue specimens were immediately fixed by 4% paraformaldehyde in phosphate-buffered saline (PBS; 137 mM NaCl, 27 mM KCl, 4.3 mM Na_2_HPO_4_, and 1.4 mM KH_2_PO_4_) and cryoembedded. The embedded tissues were vertically sectioned and the specimens were subjected to immunohistochemical detection of CD34, vascular endothelial growth factor receptor-2 (Flk-1, VEGFR-2), and VE-cadherin. For detection of CD34, Flk-1, and VE-cadherin, tissue sections were washed by PBS-T (0.1% tween in PBS). After several washes with PBS, tissue sections were permeabilized with 5% donkey serum, washed in 0.1% triton X-100 in PBS to suppress peripheral nonspecific reactivity and then incubated with anti-CD34 and anti-Flk-1 primary antibodies (Santa Cruz Biotechnology, CA, USA) overnight at 4°C. After being washed in PBS-T, sections were incubated for 2 hrs with fluorescence-conjugated antibodies (Invitrogen, CA, USA) for confocal microscopy analysis. Immunostained tissue sections were mounted with aqueous permanent mounting medium with DAPI and were examined by light microscopy and confocal microscopy. The confocal images were acquired by use of a laser confocal scanning microscope (TCS SP5/AOBS/Tandem, Leica, Germany) at Korea Basic Science Institute, Gwangju Center.

### 2.3. Flow Cytometric Analysis

Isolated cells from a portion of each penis were harvested, washed with PBS, and resuspended in fluorescence-activated cell sorter (FACS) washing buffer (1% fetal bovine serum and 0.1% sodium azide in PBS). Cells were first blocked with 0.5% (v/v) normal goat serum for 15 min at 4°C and stained with FITC-conjugated anti-CD34, PE-conjugated anti-Flk-1, and anti-VE-cadherin antibodies for 30 min at 4°C. The stained cells were analyzed by using a FACSCalibur flow cytometer (Becton Dickinson, CA, USA).

### 2.4. Statistics

All results are expressed as means ± SDs of the indicated number of experiments. Statistical significance was estimated by using *t*-tests, and the differences were compared with regard to statistical significance by one-way ANOVA, followed by Bonferroni's post hoc test. A *P* value of 0.05 was considered significant.

## 3. Results

### 3.1. Identification of Resident EPCs in the Corpus Cavernosum

**Figure 1:**
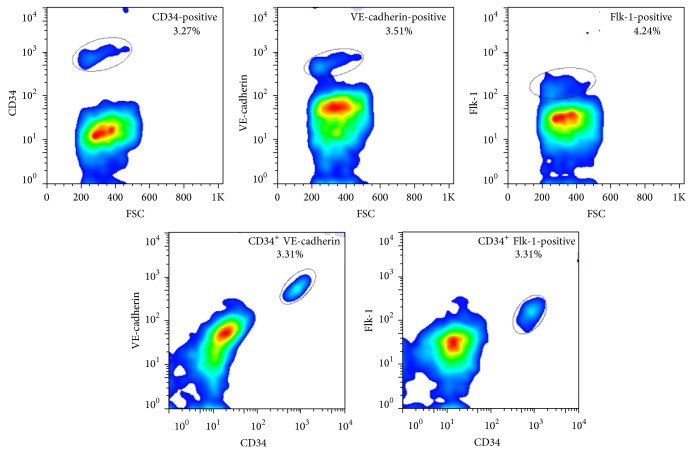
Flow cytometric analysis of EPC-specific marker expression in the rat corpus cavernosum. To determine the existence of resident EPCs in rat corpus cavernosum, CD34 and Flk-1 were used as EPC markers and double-positive cells (EPCs) were measured by flow cytometry. As shown in the figure, EPCs made up 3.31% (CD34^+^/Flk-1^+^) and 3.31% (CD34^+^/VE-cadherin^+^) of the rat penile corpus cavernosum. The results are representative of four individual experiments. EPC: endothelial progenitor cell. ^*^ *P* < 0.05 versus normal.

We assessed the distribution of penile EPCs by immunostaining and FACS analysis. Isolated cells from the penis were stained with anti-CD34, anti-Flk-1, and anti-VE-cadherin antibodies for flow cytometric analysis. CD34 is hematopoietic stem/progenitor cell marker, and Flk-1 and VE-cadherin are endothelial cell markers. Accordingly, CD34^+^/Flk-1^+^ and CD34^+^/VE-cadherin^+^ cells indicate EPCs. The results showed that EPCs made up approximately 3.31% (CD34^+^/Flk-1^+^) and 3.31% (CD34^+^/VE-cadherin^+^) of the total penile cell population (Figure 1).

### 3.2. Localization of Resident EPCs in the Corpus Cavernosum

**Figure 2:**
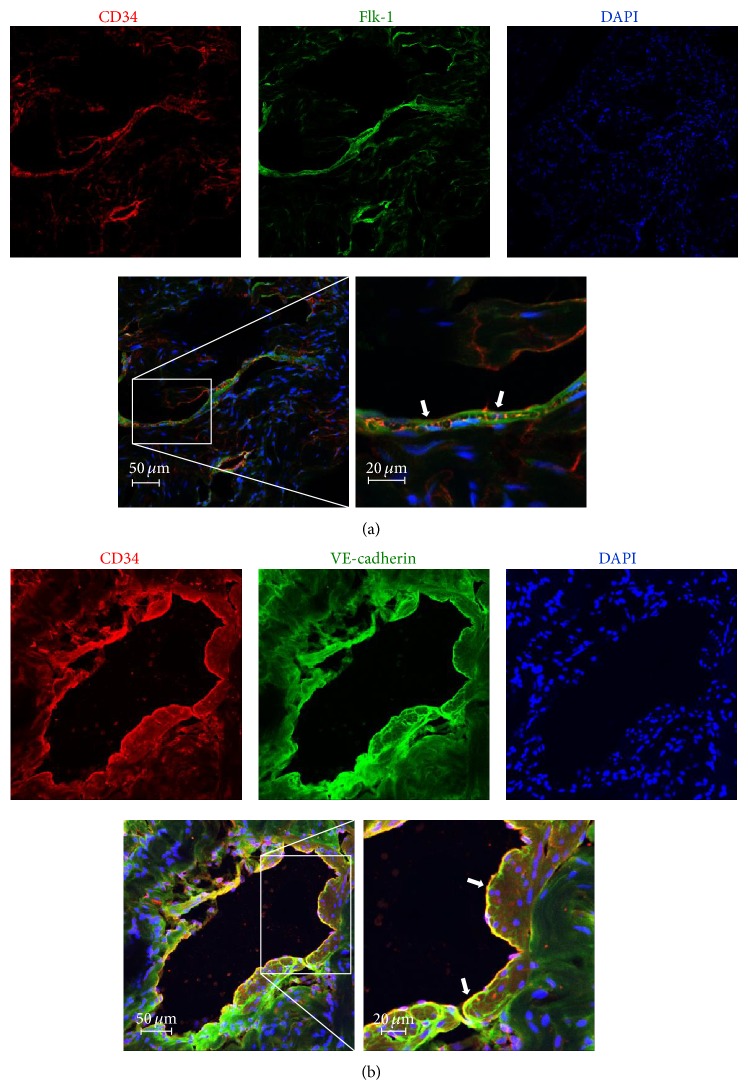
Double immunofluorescence detection of CD34, Flk-1, and VE-cadherin in the rat corpus cavernosum. Merged images represent the imposition of the staining for each single antigen. CD34/Flk-1 and CD34/VE-cadherin double-positive cells were localized in the cavernosal sinusoidal endothelial layer (arrow). The immunofluorescent images were acquired by using confocal microscopy. Data are representative of three or more independent experiments.

In addition, localization of the EPCs was identified by immunohistochemistry with anti-CD34, anti-Flk-1, and anti-VE-cadherin antibodies. To exclude circulating EPCs, we eliminated the blood by perfusion of saline through the abdominal aorta. As shown in Figures 2(a) and 2(b), CD34/Flk-1 double-labeled and CD34/VE-cadherin double-labeled cells were localized in the cavernous sinusoidal endothelial space. These data indicated that resident EPCs exist in the endothelial layer of the corpus cavernosum.

## 4. Discussion

In erectile tissue, the endothelium lining the vascular bed and the trabeculae in the penis plays a critical role in erectile function [9]. EPCs are important in vascular endothelium regeneration and repair of endothelial injury in vascular beds. Traish and Galoosian reported that putative EPCs isolated from human peripheral blood express two antigens, CD43 and Flk-1 [9]. In the present study, we used these markers to verify the existence and localization of EPCs in the corpus cavernosum in rats.

We found that EPCs made up approximately 3.31% of the penile cell population and were present in the corpus cavernosum and tunica albuginea. CD34 and Flk-1 double-stained EPCs were detected in the region of the cavernosal sinusoidal endothelium. EPCs are characterized as CD34^+^ hematopoietic progenitors from populations that expresses CD34, CD133, and VE-cadherin as well as Flk-1 markers [10], whereas circulating EPCs express a variety of markers and are characterized as CD34^+^/Flk-1^+^/VE-cadherin^+^ [10]. CD34 and Flk-1 are expressed in both early EPCs and late EPCs. Coexpression of CD34 and Flk-1, or CD34 and VE-cadherin, has been widely used to detect EPCs [9, 11]. In the present study, we confirmed the presence of CD34 and Flk-1 or CD34 and VE-cadherin coimmunoreactive EPCs in the corpus cavernosum by using flow cytometry. Klein et al. previously reported the existence of resident “side population” cells that have phenotypic and functional progenitor cell properties in the tunica media of adult mice aortas [12]. They found that vascular progenitor cells make up 6% of normal arterial wall.

Zengin et al. reported the existence of EPCs in the vascular wall, localized between the smooth muscle and adventitial layer of the adult vascular wall [6, 9]. They suggested that this vascular zone in the vascular wall may serve as a source of progenitor cells for postnatal vasculogenesis. The existence of resident EPCs has also been defined in tissues such as heart and corpus luteum [1–3]. Ovarian samples comprising corpora lutea from adult cows expressed mRNA of CD133, CD34, and Flk-1, and CD34 and Flk-1 double-positive cells were detected in the media of the arterial vessel wall. In the current study, CD34^+^/Flk-1^+^ and CD34^+^/VE-cadherin^+^ double-positive cells were localized in the cavernosal sinusoidal endothelial layer (Figures 2(a) and 2(b)). In the normal vascular wall, EPCs have been identified in the subendothelial space and vascular adventitia near the tunica media [12].

The role of EPCs in postnatal life is postulated to be the maintenance of normal vessel physiology and also contributing to vascular regeneration [13]. The presence of EPCs in the cavernosal sinusoidal endothelial layer may have potential to repair damaged endothelium. The endothelial monolayer of the corpus cavernosum plays an essential role in the vascular hemodynamics of penile erection. Any conditions that alter endothelial function, thus impairing biological activity and functional integrity, influence erectile function. Intensive studies have been performed to identify the vascular risk factors that impair penile endothelial function and that result in vasculogenic erectile dysfunction [1]. Therefore, treatment strategies for vasculogenic erectile dysfunction have focused on improving the endothelial function of the corpus cavernosum. Such strategies include oral phosphodiesterase-5 inhibitors and intracavernosal injection of vasoactive agents [1].

In the present study we aimed to identify and localize EPCs in the corpus cavernosum in a male rat model. Therefore, we did not investigate the function of the EPCs which have both stem cell like qualities and mature endothelial function. Further studies are needed to explore a functional study from isolated corpus cavernosal endothelial progenitor cells.

New treatment strategies include therapeutic angiogenesis and stem cell/gene therapy for the treatment of erectile dysfunction [14]. Angiogenic factors such as VEGF and bFGF have been tried to restore normal penile vasculature function [14, 15]. The rationale is that these angiogenic factors could replace or repair damaged endothelial cells.

In conclusion, we found the existence of EPCs in the endothelial layer of the corpus cavernosum. It is postulated that cytokines are essential for the process of differentiation of EPCs into endothelial cells. Further studies are needed to investigate the regulatory pathways of EPC regeneration by various factors, including castration, testosterone, and growth factors.
